# Neurophysiological evidence that musical training influences the recruitment of right hemispheric homologues for speech perception

**DOI:** 10.3389/fpsyg.2014.00171

**Published:** 2014-03-03

**Authors:** McNeel G. Jantzen, Bradley M. Howe, Kelly J. Jantzen

**Affiliations:** Department of Psychology, Western Washington UniversityBellingham, WA, USA

**Keywords:** musical training, musicians, language, speech processing, speech, transfer effects

## Abstract

Musicians have a more accurate temporal and tonal representation of auditory stimuli than their non-musician counterparts (Musacchia et al., 2007; Parbery-Clark et al., 2009a; Zendel and Alain, 2009; Kraus and Chandrasekaran, 2010). Musicians who are adept at the production and perception of music are also more sensitive to key acoustic features of speech such as voice onset timing and pitch. Together, these data suggest that musical training may enhance the processing of acoustic information for speech sounds. In the current study, we sought to provide neural evidence that musicians process speech and music in a similar way. We hypothesized that for musicians, right hemisphere areas traditionally associated with music are also engaged for the processing of speech sounds. In contrast we predicted that in non-musicians processing of speech sounds would be localized to traditional left hemisphere language areas. Speech stimuli differing in voice onset time was presented using a dichotic listening paradigm. Subjects either indicated aural location for a specified speech sound or identified a specific speech sound from a directed aural location. Musical training effects and organization of acoustic features were reflected by activity in source generators of the P50. This included greater activation of right middle temporal gyrus and superior temporal gyrus in musicians. The findings demonstrate recruitment of right hemisphere in musicians for discriminating speech sounds and a putative broadening of their language network. Musicians appear to have an increased sensitivity to acoustic features and enhanced selective attention to temporal features of speech that is facilitated by musical training and supported, in part, by right hemisphere homologues of established speech processing regions of the brain.

## INTRODUCTION

Research investigating the neural mechanisms involved in the processing of music and language has expanded from Bever and Chiarello’s (1974) proposed hemispheric specialization to Tallal and Gaab’s (2006) identification of similar neural areas to the evolving neuroanatomical models of Hickok and Poeppel (2000, 2007). While numerous studies have focused on specialized neural networks for the processing of either speech or music (Peretz et al., 1994; Zatorre et al., 2002; Rogalsky et al., 2011), a growing body of work has revealed that the neural mechanisms involved in the perception and processing of music overlap with those for the perception and processing of speech (Sammler et al., 2007; Wong et al., 2007; Rogalsky et al., 2011; Schulze et al., 2011). Moreover, studies have demonstrated that musical training induces neural changes resulting in enhanced speech perception in musicians (Zatorre and Belin, 2001; Zatorre et al., 2002; Bever and Chiarello, 2009). Specifically, musical training enhances language processing by altering neural networks for perception and processing of speech (Thompson et al., 2003; Schön et al., 2004; Moreno and Besson, 2006; Besson et al., 2007; Parbery-Clark et al., 2009a; Shahin, 2011). In addition to enhancing activity in speech processing areas, musicians may also engage right hemisphere music processing areas for the perception of speech. The present study examined whether alterations stemming from musical training were lateralized to traditional left language areas or extended into right hemisphere homologues for speech processing.

The perception and processing of acoustic features such as onset time and pitch are common to speech and music (Tremblay et al., 2001; Reinke et al., 2003) and are represented hierarchically in the auditory cortex. The primary auditory cortex encodes onset time and pitch, with speech sounds processed mainly in the left auditory cortex (Zatorre and Belin, 2001; Zatorre, 2002). Musicians who engage left hemisphere auditory cortex during the processing and perception of these features during tasks involved in musical training may in turn enhance their ability to perceive temporal aspects of speech sounds (Shahin, 2011). Voice-onset-time (VOT), the duration of the delay between release of closure and start of voicing (Lisker and Abramson, 1964), is one of the most important temporal acoustic cues in speech because it carries linguistically and phonetically relevant information (Ott et al., 2011) that allows us to perceive the difference between a voiced (e.g., /b/) and voiceless (e.g., /p/) stop consonant (Chobert et al., 2012). VOT is also important to the development of phonological representations (Chobert et al., 2012). Studies have demonstrated that musicians are more sensitive to and process voiceless stimuli differently than their non-musician counterparts (Chobert et al., 2011; Ott et al., 2011). However, the neural basis of this difference has not been explored.

Perceptual categorization is another important component to both speech and music. The ability to categorize musical stimuli has been shown to predict categorization of speech stimuli, suggesting that the two processes share a common cognitive mechanism (Overy, 2003; Tallal and Gaab, 2006; Patel and Iversen, 2007; Wong et al., 2007). Speech categorization relies primarily on timbral contrasts and music categorization primarily on pitch contrasts. For example, in speech the range of possible vowel sounds is a continuum, but speakers of a language learn to separate this continuum into discrete vowels. Similarly, the range of possible frequencies in music is continuous, with musicians learning to categorize these frequencies into discrete notes. Musicians have also demonstrated a more accurate temporal and tonal representation of auditory stimuli than their non-musician counterparts (Musacchia et al., 2007; Parbery-Clark et al., 2009a; Zendel and Alain, 2009; Kraus and Chandrasekaran, 2010), though the actual mechanism behind this advantage is less obvious. Moreover, their temporal representations are less susceptible to the negative effects of background noise (Parbery-Clark et al., 2009a). Musical training has also been shown to provide advantages to perceptual and attentional mechanisms for language (Strait and Kraus, 2011). Components of Patel’s (2011) OPERA hypothesis such as: Overlap, Precision, Repetition, and Attention describe how musical training might create these advantages. First there is an anatomical overlap of neural areas that process acoustic features present in both speech and music. Secondly, music requires greater precision than speech and thus places a higher demand on overlapping neural areas. Finally, musical training requires repetition therefore continually engaging these neural areas that have also been shown to be associated with focused attention. Another possibility is that corticofugal mechanisms induce short and long term plasticity resulting in a transfer of training from music to language (Kraus and Chandrasekaran, 2010; Besson et al., 2011; Chobert et al., 2012). Musicians have demonstrated an advantage over non-musicians in their ability to recognize tonal variations in non-native speech sounds (Wong et al., 2007; Cooper and Wang, 2010; Perfors and Ong, 2012). This advantage can be attributed to formal musical training that emphasizes enhanced perception of pitch and may provide them an advantage when learning speech sounds.

When considered together, this literature suggests that musical training is related to neuroplastic changes to the language network as musicians’ become more sensitive to the acoustic features critical to both speech and music. We predict that musical training enhances speech perception and discrimination in musicians by engaging right hemisphere brain regions more typically associated with music processing. This prediction reflects our broader hypothesis that music and language are processed in partially overlapping networks and that the right hemisphere components of this network are enhanced by musical training. Here, we recorded electroencephalography (EEG) to address whether musicians’ engage neural areas that are not typically associated with left hemisphere dominant language networks when discriminating between phonemes differing in voice onset time. An analysis of cortical sources revealed greater right hemisphere engagement for musicians compared to non-musicians.

## MATERIALS AND METHODS

### SUBJECTS

Twelve right-handed (evaluated using the Oldfield Handedness Inventory, Oldfield, 1971) monolingual American-English speakers who reported normal hearing were recruited from the music department and general population at Western Washington University and divided into musician and non-musician groups. Musicians (*n* = 6) were required to have at least 5 years of continuous formal musical training (*M* = 9.17 years, SD = 2.11) and all played wind instruments. Non-musicians (*n* = 6) had no musical training and had never played a musical instrument. Participants ranged in age from 19 to 22 years (*M* = 20.25 years, SD = 0.83). All procedures were conducted with written consent from participants and with the approval of the Western Washington University Human Subjects Committee.

### STIMULI

Auditory stimuli were presented at 75 dB via over-ear Sennheiser HD-595 using custom Visual Basic software that controlled the timing and added event markers to the EEG record for subsequent segmentation of individual data epochs. Four synthetic CV stimuli were created in Synthworks (Scion, R&D Inc.), with C consisting of either the voiced unaspirated /d/ or voiceless unaspirated /t/ followed by a 215 ms vowel /**α**/. The duration of the voiced consonant was 100 ms and the voiceless consonant was 45 ms.

### PROCEDURE

In keeping with previous studies (Belin et al., 2000; Parbery-Clark et al., 2009a,b; Kerlin et al., 2010) we used a dichotic listening task. In four tasks participants were presented with different speech sounds in each ear and instructed to attend to a specific aural location or to listen for a specific speech sound. The four tasks were (1) D Sound (2) T Sound (3) Right Ear and, (4) Left Ear. In the D and T Sound tasks, subjects were instructed to focus their attention on the /d/ and /t/ sound respectively regardless of the ear of presentation. In the Right and Left Ear tasks subjects were instructed to focus their attention on their right or left ear respectively. To minimize voiceless dominance, stimuli were onset-aligned rather than aligned to the noise burst and dichotic pairs consisted of all possible VOT (voiced/voiceless) combinations. For each task 120 stimulus pairs were presented consisting of 60 instances of each of the two combinations. In each condition, stimuli were shuffled in a pseudo-randomized order. The condition order was randomized for every subject. We collapsed our measures of performance and event related potentials (ERP) across all dichotic listening tasks. The full analysis of the individual behavioral tasks will be presented in a separate report.

### EEG DATA ACQUISITION

Electroenchapalographic signals were recorded continuously from 64 Ag/AgCl active electrodes (Active 2 System, Biosemi, Amsterdam, Netherlands) mounted in an elastic headcap according to a 10–10 configuration (Chatrian et al., 1985). Signals were conducted using a saline-based conductive gel (Signa Gel) and all offsets were maintained below 20 uV. Unreferenced signals were amplified and digitized at 512 Hz using Biosemi Active Two amplifiers and acquisition software. Although electromyography activity was not recorded, all participants were given specific instructions to refrain from moving during the experiment and participants were monitored for evidence of unintended or unconscious movements. The experimenters did not observe any overt movement.

### ERP ANALYSIS

**FIGURE 1:**
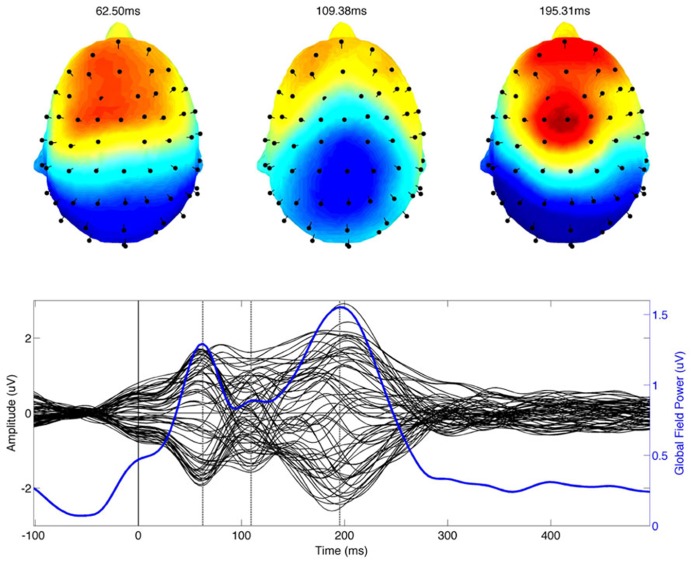
**The combined grand average of musicians and non-musicians of the event related potential is shown in the bottom panel.** The time series from each of the 64 recording electrodes are shown in black on the same axis. The global field power computed using all electrodes is shown in blue. The time of identified ERP (P50m, N100, and P200) components is indicated by vertical dotted lines. The scalp topography of each ERP component is shown in the top row of the figure.

Data processing and visualization was accomplished using the EEGLab toolbox running under Matlab 7.0. Continuous data from each participant were referenced to the average potential of all electrodes. For all conditions EEG epochs were extracted in the interval from -100 to 500 ms around the onset of the stimulus. Epoched data was bandpass filtered between 1 and 20 Hz. Trials containing large signals exceeding 100 uV were automatically identified, manually inspected and rejected if they were judged to contain artifacts. Trials were also inspected for EMG contamination. Eye blink and eye movement artifacts were identified and removed in EEGLab (Delorme and Makeig, 2004) using an established independent component analysis (ICA) approach (Jung et al., 2000). For each participant, epoched data were linearly unmixed or decomposed into 64 maximally independent components. Eye blink and other artifact related components were identified based on their characteristic spatiotemporal pattern. The contribution of these components was set to zero and the data were projected back into the original sensor space. This procedure removes the contribution of the artifact without altering the evoked brain response thereby elimination the need to discard large numbers due to excessive blinking (Jung et al., 2000). The resulting trial epochs were used to compute the average evoked response for each participant. A qualitative description of ERP components was based on visual inspection of the grand average ERP, the associated scalp distributions and the global field power of the grand average (see **Figure 1**).

Because our primary hypothesis predicts group differences in ERP source generators for musicians and controls, we performed analysis on cortical sources estimated using standardized low-resolution brain electromagnetic tomography (sLORETA) (Pascual-Marqui et al., 1994, 2002). Before comparing the source distributions, we first identified time points of interest by statistically comparing the ERP time series from the musician and control groups. A non-parametric permutation test using 10000 permutations [as implemented in EEGLAB function “statcond” (Delorme, 2006)] was used to compare ERP of musicians and non-musicians averaged across four electrode montages reflecting our hypothesis that language related activity would originate from left and right inferior frontal and posterior temporal regions. The electrodes included in the montage over the inferior frontal region were AF3, AF7, F5, F7, FC5, and FT7 on the left and AF4, AF8, F6, F8, FC6, and FT8 on the right. For the temporal parietal regions we selected electrodes CP1, CP3, CP5, P1, P3, and P5 on the left, and CP2, CP4, CP6, P2, P4, and P6 on the right. A separate permutation test was run to compare ERP amplitude between musicians and non-musicians for each montage and time points between 3 to 300 ms. This time range was selected because it represents the period during which early auditory processing occurs. We controlled for multiple comparisons using false discovery rate (Benjamini and Yekutieli, 2001) to achieve a corrected *p* < 0.05. Analysis of the cortical sources was performed at intervals showing significant differences between groups.

To obtain estimates of cortical generators, we applied sLORETA to the average scalp-recorded electric potential distribution of each participant to compute the distribution of current density on a template brain in Talairach coordinates (Talairach and Tournoux, 1988). A parametric two sample *t*-tests was computed on the amplitude normalized and log-transformed sLORETA images (Thatcher et al., 2005). Tests of skewness confirmed that the log transform generated images that approximated a normal distribution (mean skewness = -0.40) for all participants. Multiple comparisons (voxels = 6239) were controlled using false discovery rate with a corrected *p* < 0.05. The corrected t threshold in the statistical parametric map was 6.43. The location of cortical regions in which voxels exceeded this threshold was determined using Talairach atlas information available in the LORETA software.

## RESULTS

### BEHAVIORAL RESULTS

Discrimination performance of musicians and non-musicians was measured across all stimuli and tasks. Mean correct responses (musicians, *M* = 256; non-musicians, *M* = 264) and a between subjects *t*-test performed using SPSS showed that musical training did not improve the ability to detect correct stimuli based upon differences in voice onset time [*t*(10) = 0.470, *p *= 0.649].

### ERP ANALYSIS

Three dominant component peaks were observed in the grand average ERP data and the global field power (**Figure 1**; lower panel). The scalp distribution of the time of the component peaks corresponds well with the expected P50-N1-P2 complex (**Figure 1**). The latency of the peak amplitude of the three components was 62.5, 109.38, and 195.31 ms respectively.

**FIGURE 2:**
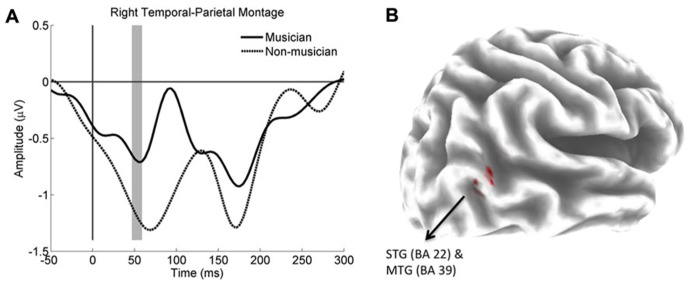
**(A)** Group average ERPs for the right temporal parietal montage. Compare to non-musicians (dotted line), ERP for musicians (solid line) was significantly more positive within a contiguous window 46.8 to 54.6 ms (gray shading). **(B)** Shows the significant difference between the sLORETA images of the musicians and non-musicians. Musicians had significantly greater activity in the right superior temporal gyrus (STG) and middle temporal gyrus (MTG). Differences are shown in red on a partially inflated template brain.

Non-parametric tests revealed significant differences in the right temporal parietal montage at three consecutive time points from 46.8 to 54.6 ms, at the approximate time of the P50 ERP component. Activity was significantly more positive for the musicians than the non-musicians. Musicians also showed significantly greater activity in the right superior temporal gyrus (STG; BA 22) and middle temporal gyrus (MTG; BA 39) during the time interval from 46.8 to 54.6 ms. Voxels showing significant differences are shown in **Figure 2**. The maximum *T* value of 7.46 was Talairach location at *X* = 50, *Y* = -57, *Z* = 17 in Brodmann area 22.

Although substantial differences in ERP amplitude were also observed at times corresponding approximately to the N1 (~100 ms) and P2 (~175 ms), the differences were not significant after correcting for multiple comparisons.

## DISCUSSION

The purpose of this study was to examine how musical training affects the neural organization and representation of speech. We were specifically interested in the early processing of acoustic components of the speech signal. Our hypothesis was that musical training induces alterations to neural areas associated with both music and language, thereby modifying the language network of musicians. In support of our hypothesis, ERP differences between musicians and non-musicians were observed around the time of the P50 response. Analysis of cortical sources revealed greater activity in right hemisphere for musicians during this time frame at the posterior junction of the superior and middle temporal gyri.

Right hemisphere activity in MTG and STG in musicians may reflect enhanced processing of speech sounds. The P50 originates in the STG (Eggermont and Ponton, 2002) and reflects early auditory neurophysiological processes (Ott et al., 2011), in particular, early speech-specific processing of phonemes and syllables (Dehaene-Lambertz et al., 2005). Previous research has shown regions in the superior temporal lobes responsive to perceiving speech sounds (Wise et al., 1991; Mummery et al., 1999; Belin et al., 2000; Binder et al., 2000; Scott et al., 2000). More specifically, the STG and superior temporal sulcus (STS) are sensitive to complex spectrotemporal information (Zatorre et al., 1992; Binder et al., 2000; Scott et al., 2000; Poeppel, 2003). It had been presumed that activity in the temporal lobe regions was lateralized to left dominant STG and STS for phonetic and phonological speech perception (Liebenthal et al., 2005; Möttönen et al., 2006; Hickok and Poeppel, 2007). The major finding of the present study is greater activation of right MTG and STG for musicians. Traditionally, MTG has been implicated in lexical-semantic processing (Demonet et al., 1992; Vandenberghe et al., 1996; Binder et al., 1997; Dronkers et al., 2004). However, more recent research implicates the MTG in phonemic discrimination tasks (Ashtari et al., 2004) and, contrary to previous research, right hemisphere sensitivity to phonemic information may not be limited to a lexical content (Wolmetz et al., 2011). Moreover, activation of the right MTG region at the time of the P50 component of the ERP is compatible with a putative role in encoding of early acoustic features (Schneider et al., 2002).

Another possibility is that the activity we report in STG at 50 ms reflects an enhanced role of right hemisphere for selective attention (Shahin, 2011). The P50 amplitude is thought to reflect top down attentional change and processes associated with working memory, such as our ability to selectively attend to salient stimuli and inhibit processing of irrelevant information (Light and Braff, 2003; Beratis et al., 2009; Sur and Sinha, 2009). Selective attention mechanisms are necessary in the processing of noisy auditory scene situations (Parbery-Clark et al., 2009b; Kerlin et al., 2010) such as the dichotic listening task used in the present study. If selective attention abilities are enhanced in musicians, it would impact how relevant and irrelevant signals are organized in working memory (Sreenivasan and Jha, 2007) and possibly promote relevant acoustical signal intensity while simultaneously suppressing interfering noise (Kerlin et al., 2010). Musicians’ focus on and direct their attention to small changes in acoustical features such as pitch and onset time, thereby developing an acute processing of spectrotemporal acoustical information (Schneider et al., 2002; Marie et al., 2012). This enhanced representation of acoustical information facilitates acoustical feature binding and analysis of the acoustic scene (Treisman and Gelade, 1980; Shinn-Cunningham and Best, 2008), particularly at P50 (Shahin, 2011). Musicians improved performance over non-musicians in auditory tasks requiring focused attention (Strait et al., 2010) may result from improved auditory scene analysis skills that have been shaped by selective attention mechanisms via enhanced acuity to acoustical features (Shahin, 2011). Auditory scene analysis is also influenced by cognitive mechanisms associated with working memory and target detection, both of which are required by musicians when attentively listening to music (Janata et al., 2002) and may lead to improved concurrent sound segregation (Shahin, 2011). Furthermore, segregation of sound is important during dichotic listening tasks and may account for musicians’ recruitment of the right STG.

Previous research examining differences in the processing and analysis of acoustic features between musicians and non-musicians show both functional and structural alterations (Pantev et al., 1998, 2003; Magne et al., 2003; Moreno and Besson, 2005; Chartrand and Belin, 2006; Moreno and Besson, 2006; Baumann et al., 2008) even after only a short exposure to musical training (Magne et al., 2006; Moreno et al., 2009). Taken together, these studies elucidate musicians’ expertise at processing spectrotemporally complex acoustic information. The present study complements this growing body of work by demonstrating recruitment of right hemisphere in musicians for discriminating speech sounds. The increased activation of speech related areas with respect to P50 demonstrate a putative broadening of the speech processing network induced by musical training. We believe these results may reflect enhanced selective attention and increased sensitivity to acoustic features of speech that is facilitated by musical training and supported, in part, by right hemisphere homologues of established speech processing regions of the brain.

In addition to the P50, evidence supports enhancement of later ERP components resulting from musical training. P50, N1, and P2 components have been found to be important for auditory analysis and coding of low-level acoustical features and representing higher-level complex spectrotemporal sound features (Sharma and Dorman, 1999; Steinschneider et al., 1999, 2005; Sharma et al., 2000; Zaehle et al., 2007; Shahin, 2011; Ott et al., 2011). Moreover, earlier occurring components such as the P50 may influence later components such as the N1 and P2 (Gilbert et al., 2001). Previously, N1 (Sharma et al., 2000; Zaehle et al., 2007; Ott et al., 2011) and P2 amplitudes (Tremblay et al., 2001; Tremblay and Kraus, 2002) differed either for voiced versus voiceless stimuli or in musicians versus non-musicians. Although preliminary analysis of the N1 and P2 components in the present data revealed differences between musicians and non-musicians, these findings did not survive statistical correction for multiple comparisons. Thus although the current work supports that musical training influences early acoustic processing, subsequent studies may reveal more subtle differences in later processing as well.

The lack of a behavioral advantage for musicians may stem from the difficulty of the dichotic listening task. Both groups performed at just better than chance level suggesting that they found the task very difficult. Although the dichotic listening task was to ensure attentive auditory processing, future studies may consider altering task demands to be more sensitive to potential performance differences. Another potential limitation of the present study is the small sample size. Although, recent studies that performed between subject experiments with a sample size of 10 or fewer per group also report significant and robust neural differences (Schön et al., 2004; Magne et al., 2006; Besson et al., 2007; Santos et al., 2007), it is important to consider the relatively small number of participants in our study when interpreting these results more broadly.

The role of the right hemisphere and its contribution to speech perception is still a matter of debate. Obleser and Eisner (2009) argue that the right hemisphere plays no role in speech perception. Similarly, left temporal lateralization is supported by a review of studies in which contrasts were related to phoneme-specific processing (Wolmetz et al., 2011). In contrast, however, Hickok and Poeppel (2007) have strongly argued for inclusion of the right hemisphere based upon bilateral activation during speech perception tasks. The present results support this latter position and suggest the possibility that right hemisphere MTG/STG activation in musicians during discrimination of speech consonants differing in VOT results from musical training induced sensitivity and enhanced selective attention to temporal features within the speech signal.

## Conflict of Interest Statement

The authors declare that the research was conducted in the absence of any commercial or financial relationships that could be construed as a potential conflict of interest.
